# Epstein–barr virus vaccines

**DOI:** 10.1038/cti.2014.27

**Published:** 2015-01-23

**Authors:** Jeffrey I Cohen

**Affiliations:** 1 Medical Virology Section, Laboratory of Infectious Diseases, National Institute of Allergy and Infectious Diseases, National Institutes of Health, Bethesda, MD, USA

## Abstract

Epstein–Barr virus (EBV) is the primary cause of infectious mononucleosis (IM) and is associated with epithelial cell malignancies such as nasopharyngeal carcinoma and gastric carcinoma, as well as lymphoid malignancies including Hodgkin lymphoma, Burkitt lymphoma, non-Hodgkin lymphoma and post-transplant lymphoproliferative disorder. EBV vaccines to prevent primary infection or disease, or therapeutic vaccines to treat EBV malignancies have not been licensed. Most efforts to develop prophylactic vaccines have focused on EBV gp350, which is the major target of neutralizing antibody. A single phase 2 trial of an EBV gp350 vaccine has been reported; the vaccine reduced the rate of IM but not virus infection. The observation that infusion of EBV-specific T cells can reduce disease due to Hodgkin lymphoma and nasopharyngeal carcinoma provides a proof of principle that a therapeutic vaccine for these and other EBV-associated malignancies might be effective. Most therapeutic vaccines have targeted EBV LMP2 and EBV nuclear antigen-1. As EBV is associated with nearly 200 000 new malignancies each year worldwide, an EBV vaccine to prevent these diseases is needed.

Over 95% of adults are infected with Epstein–Barr virus (EBV); most infections occur in young children and are asymptomatic or cause nonspecific symptoms.^1^ EBV is the primary cause of infectious mononucleosis (IM) and is associated with a number of B lymphocyte and epithelial cell malignancies. In a recent study, 37% of students entering a college in the United States were EBV seronegative, 46% of them seroconverted during 3 years of college and 77% of those who seroconverted developed symptoms of IM.^2^ Although IM is a self-limited illness, 20% of patients have fatigue that persists for 2 or more months and 10% have fatigue for 6 or more months.^3^ It is estimated that 1% of the patients with IM have severe neurological, bone marrow or liver disease. IM is the most common cause of lost time for new Army recruits. Therefore, IM is not always a benign disease, and a vaccine that could prevent the disease would be useful. Such a vaccine might be given at ages 11–12 (at the same time as the human papillomavirus vaccine) to children in developed countries. EBV infection occurs at a younger age in developing countries and in certain racial/ethnic groups in developed countries,^4^ therefore vaccination would need to be given at a younger age in these groups.

EBV is associated with Burkitt and Hodgkin lymphoma in otherwise healthy persons and with immunoblastic, Burkitt and Hodgkin lymphoma in patients with AIDS. About 40% of Hodgkin lymphomas are EBV positive in persons in affluent countries, whereas 80% of these lymphomas are EBV positive in persons in developing countries. About 1 in 800 persons with EBV-positive IM in Scandinavia develop Hodgkin lymphoma, with a median time from IM to lymphoma of 4 years.^5^ In contrast, the overall rate of Hodgkin lymphoma is ~1 in 40 000 persons in Europe.^6^ Therefore, a vaccine that reduces IM might reduce the rate of Hodgkin lymphoma. About 85% of Burkitt lymphomas in Africa are EBV positive, whereas 15% of these lymphomas in the United States are EBV positive. In Africa, 50% of the children are infected before 1 year of age, hence a vaccine would need to be given to infants.

Nearly all anaplastic nasopharyngeal carcinomas contain EBV DNA in the tumor cells. About 9% of gastric carcinomas are associated with EBV and 90% of gastric lymphoepitheliomas are EBV positive. The peak incidence of nasopharyngeal carcinoma and gastric carcinoma is 50–59 years and 50–70 years, respectively.

EBV is associated with lymphoproliferative disease in patients with congenital or acquired immunodeficiencies.^7, 8^ These include patients with severe combined immunodeficiency, patients with AIDS, or recipients of organ or bone marrow transplants. These patients have impaired T-cell immunity and are unable to control the proliferation of EBV-infected B cells. EBV post-transplant lymphoproliferative disorder usually occurs during the first year after hematopoietic stem cell transplantation. The rate of post-transplant lymphoproliferative disorder is 25–30-fold higher for persons who are EBV seronegative before transplant, compared with those who are seropositive before transplant.^9^ This implies that a vaccine that prevents or possibly reduces the severity of primary infection might reduce the rate of post-transplant lymphoproliferative disorder. The EBV DNA level in the blood is predictive of the development of EBV post-transplant lymphoproliferative disorder. Rituximab, which is given when the EBV DNA level in the blood is rising before the onset of lymphoproliferative disease, usually results in a marked decline in the viral load and reduces the risk of the disease.

EBV is also associated with autoimmune diseases, including multiple sclerosis. Primary EBV infection^10^ as well as IM^11^ is associated with an increased risk of multiple sclerosis. The mean time between EBV infection and development of multiple sclerosis was estimated to be about 6 years in one study.^10^

Effective vaccines have been developed for several herpesviruses. The live attenuated Oka vaccine prevents varicella and reduces the rate of zoster, which is due to the reactivation of the virus. This vaccine does not prevent infection with varicella-zoster virus. Vaccines have also been developed for Marek's disease and herpesvirus saimiri, which are herpesviruses that cause malignant disease in chickens and monkeys, respectively.

## EBV glycoproteins

EBV encodes several viral glycoproteins that are present on the surface of the virion including glycoprotein gp350, gB, gH, gL, gp42 and BMRF2.^12^ gp350 has been studied most intensively as a vaccine immunogen. It is the most abundant glycoprotein on the surface of virions and virus-infected cells. The glycoprotein binds to CD21 (also known as CR2 or C3d) on the surface of B lymphocytes, which results in endocytosis of the virion into cells. The amino-acid sequence of gp350 is highly conserved among different strains and shares 97% amino-acid identity between type 1 and 2 EBV.^13^ The amino portion of gp350, which includes the amino acids important for binding to CD21, is more highly conserved than the carboxyl portion of the glycoprotein.^14, 15^ A potent neutralizing antibody, 72A1 that recognizes gp350, neutralizes type 1 and 2 EBV strains.^16^ Of all the EBV glycoproteins, gp350 is the principle target for antibodies that neutralize infection of B cells,^17, 18, 19^ and the glycoprotein is a target for antibody-dependent cellular cytotoxicity,^20, 21^ CD4 T cells,^22, 23^ and CD8 T cells.^24^

Other EBV glycoproteins are also targets of the neutralizing antibody. Monoclonal antibody to gp42 inhibits B cell infection (Li *et al.*^25^), whereas monoclonal antibodies to gH/gL^25^ or to BMRF2^26^ inhibit EBV infection of epithelial cells. Although monoclonal antibody to gB has not yet been shown to neutralize EBV infection, gB is required for fusion with epithelial cells and B cells.^27^ EBV-neutralizing antibody titers in humans correlate more closely with antibody titers to gp350 than with antibody titers to gp42.^28^

## Non-human primate EBV vaccine trials

The first vaccine shown to protect animals from EBV-associated lymphoma was a gp350 vaccine. Intraperitoneal inoculation of cotton top tamarins with high titers of EBV results in the development of B-cell lymphomas that contain EBV DNA, and express the same viral proteins and have a similar histology to that seen in lesions from EBV lymphoproliferative disease in humans. Epstein *et al.*^29^ first showed that intraperitoneal vaccination with membranes isolated from EBV-infected cells or gp350 purified from infected cells inserted into liposomes protected cotton top tamarins from EBV lymphoma after challenge with the virus. Subsequent studies using purified or recombinant gp350 given with ISCOMs (immunostimulating complexes), muramyl dipeptide in squalene, or alum protected cotton top tamarins from lymphoma (Table 1). In addition, adenovirus or vaccinia virus expressing gp350 also protected these animals. In all but two reports neutralizing antibody to EBV was detected. In these two papers, animals receiving recombinant adenovirus^30^ or recombinant vaccinia virus WR (Western Reserve) strain^31^ failed to produce neutralizing antibodies, but were protected from lymphomas, whereas in one of these studies the animals receiving a different recombinant vaccinia virus-Wyeth strain^31^ did not develop neutralizing antibodies and all developed lymphomas. The observation that some vaccines are protective, even in the absence of neutralizing antibody, suggests that gp350 vaccines induce antibodies with other activities, such as antibody-dependent cellular cytotoxicity or T-cell responses that are protective. Subsequent experiments showed that cotton top tamarins that recovered from EBV challenge developed EBV-specific CD8 T cells.^32^ The cotton top tamarin model of EBV has several limitations: the animals are an endangered species, they are not naturally infected with the virus, the virus does not establish a latent infection in the animal's B cells (except for the tumors), lymphomas are only induced with parenteral (not oral) challenge with high doses of virus, and the animals have very limited major histocompatibility complex alleles.

Rhesus lymphocryptovirus (LCV) has also been used as a model for EBV infection and vaccination. Rhesus LCV is a naturally occurring virus that reproduces most, if not all, of the features of EBV. Infected animals shed virus from their throat, are latently infected, and the virus is associated with lymphomas in immunocompromised animals.^33^ All of the genes in EBV have homologs in rhesus LCV and most of the functions of the corresponding proteins are conserved. Sashihara *et al.*^34^ and colleagues compared three different vaccines in rhesus monkeys; soluble rhesus LCV, a virus-like replicon particle expressing rhesus LCV gp350 and virus-like replicon particles expressing rhesus LCV gp350, EBV nuclear antigen 3A (EBNA-3A) and EBNA-3B. Animals vaccinated with soluble gp350 had the highest gp350 antibody titers, whereas animals vaccinated with virus-like replicon particles expressing gp350, EBNA-3A and EBNA-3B developed rhesus LCV-specific CD4 and CD8 T-cell responses. Animals that received soluble gp350 showed the lowest rate of infection after challenge with rhesus LCV. Surprisingly, of the vaccinated animals that became infected after challenge, those vaccinated with soluble gp350 had the lowest level of rhesus LCV DNA in the blood 2 years after challenge. Since EBV DNA levels in the blood are a risk factor for development of EBV post-transplant lymphoproliferative disorder, vaccination with soluble gp350 might reduce the rate of post-transplant lymphoproliferative disorder. These results also suggest that a prophylactic vaccine targeting gp350 might be more effective than one targeting EBNA-3 proteins to reduce the incidence of infection and lower the viral load in persons who do become infected.

## Human EBV vaccine trials

EBV gp350 has been used to vaccinate humans against the virus (Table 2). Gu *et al.*^35^ vaccinated adults, children and infants in China with a single dose of vaccinia virus expressing gp350 under the 11 K vaccinia virus promoter. Although EBV-seropositive and vaccinia virus-seropositive adults had no increase in the level of EBV antibody, EBV-seropositive, vaccinia virus-seronegative children had an increase in the titer of EBV-neutralizing antibody. Nine EBV-seronegative, vaccinia virus-seronegative children aged 1–3 were vaccinated and all developed EBV-neutralizing antibody. Although 10 of the 10 unvaccinated control children became infected with EBV during 16 months of follow-up, only 3 of the 9 vaccinated children were infected. Though encouraging, vaccinia virus is unlikely to be accepted as a vaccine vector owing to its potential side effects.

Phase I/II studies of recombinant gp350 grown in Chinese hamster ovary cells showed that the vaccine induced neutralizing antibodies in humans.^36^ Comparison of subjects receiving 50 μg of soluble gp350 in no adjuvant, in alum or in alum/monophosphoryl lipid A showed that subjects receiving the vaccine in alum/monophosphoryl lipid A had the highest levels of EBV antibody based on enzyme-linked immunosorbent assay (ELISA) and EBV-neutralizing antibody assays. One subject developed headache, meningismus and polyarthritis after the second dose of vaccine; all symptoms resolved within 2 months. A phase II, randomized, double-blind placebo-controlled trial of soluble gp350 was performed in 181 EBV-seronegative students.^37^ Subjects received either three doses of soluble gp350 (50μg) in alum/monophosphoryl lipid A adjuvant or placebo (alum alone) at 0, 1 and 5 months. The vaccine induced gp350 ELISA antibody titers in 99% of the subjects when measured 1 month after the last dose of vaccine. Subjects retained anti-gp350 antibody titers for >18 months. About 70% of persons had positive competition ELISA assay titers, a surrogate for neutralizing antibody titers, at 6 months after the first vaccine dose. The vaccine reduced the rate of IM by 78% in the vaccinated subjects but did not prevent virus infection. These results suggest that a vaccine should be able to reduce the disease associated with EBV, but not necessarily prevent infection.

A similar vaccine was given to 16 EBV-seronegative children who were awaiting kidney transplantation.^38^ Subjects received three or four doses of soluble gp350 (12.5–25 μg) in alum. At 26 weeks after transplant, the peak level of EBV DNA in blood was similar in those who were vaccinated and those who had not been vaccinated, and was slightly higher than those who were EBV seropositive but had not been vaccinated. Only 4 of the 13 vaccinated subjects developed EBV-neutralizing antibody and these levels declined rapidly. Four vaccine recipients became asymptomatically infected with EBV and one developed post-transplant lymphoproliferative disorder. The failure of this vaccine to induce a potent immune response may have been due to the patient's immunosuppressed state associated with renal insufficiency, the relative low dose of gp350 used and the type of adjuvant used.

A different approach to an EBV vaccine involves the use of peptides to induce T-cell immunity to an EBV latency-associated protein. Elliott *et al.*^39^ vaccinated eight seronegative HLA B*08:01 subjects with 5 μg of an EBNA-3A peptide with tetanus toxoid in an oil and water emulsion, two with 50 μg of the peptide in the adjuvant and four with placebo. Of the nine subjects who were evaluated eight developed T-cell responses to the EBNA-3A peptide after vaccination. At 2–12 years after vaccination, four of the eight subjects who received low dose peptide seroconverted and none developed IM. One of the two who received the high dose peptide vaccine seroconverted and the subject may have had a mild case of IM. Two of the four subjects who received placebo became infected with EBV and one of the two developed IM. No adverse events were noted.

## An alternate approach for an EBV vaccine

Ruiss *et al.*^40^ described the production of virus-like particles in which several EBV proteins-EBNA-2, latent membrane protein 1 (LMP1), EBNA-3A, EBNA-3B, EBNA-3C and BZLF1- were either functionally inactivated or deleted. In addition, the viral genome lacked the TR packaging element, which is required for packaging viral DNA; therefore, the virus-like particles did not contain detectable EBV DNA. Vaccination of mice with these virus-like particles induced EBV-neutralizing antibody and cellular immune responses detected by interferon-γ enzyme-linked immunospot assays in splenocytes from the mice. Although this approach is promising, it is unclear whether the vaccine would be practical to manufacture and whether the cell line used to produce the virus-like particles would be an acceptable substrate for use in humans.

## Possible outcomes of an EBV vaccine

It is not certain that an EBV vaccine could induce immunity that protects from infection. The observation that healthy persons can be infected with EBV types 1 and 2^41^ or swarms of viruses with different sequences^42, 43^ suggests that reinfection may occur. The varicella vaccine, which is licensed to protect children from disease due to another herpesvirus-varicella-zoster virus does not protect against infection.^44^ The varicella vaccine is a live attenuated vaccine containing all of the varicella-zoster virus proteins and it seems unlikely that an EBV vaccine, which would have to contain a more limited set of viral proteins (since EBV expresses several oncogenic proteins), would be more effective.

A vaccine that does not prevent infection might still reduce disease as was evidenced by the phase 2 clinical trial showing that a gp350 vaccine lowered the rate of IM.^37^ Such a vaccine might reduce the virus load after infection resulting in a lower level of virus in the blood over time (also known as the virus set point), as was observed with the gp350 rhesus LCV vaccine.^34^ Since the EBV load in blood is a predictor of post-transplant lymphoproliferative disorder^45, 46^ a lower viral set point might reduce the risk of EBV malignancies.

## Future trials of EBV prophylactic vaccines

As a phase 2 trial showed that a gp350 vaccine reduces the rate of IM, it seems logical to perform a phase 3 trial to confirm this finding so that such a vaccine could be licensed. It would be important to collect sera after vaccination and carefully measure the titer of neutralizing antibody, preferably using an assay that directly measures the ability of the antibody to prevent virus infection or transformation of B cells. This might allow one to determine whether neutralizing antibody is a correlate of protection against IM. If neutralizing antibody does not correlate, then sera should be tested for other activities including antibody-dependent cellular cytotoxicity, as well as the subtype of IgG induced in different subjects, to look for alternate correlates of protection. In addition, the level of EBV DNA in the blood should be measured in subjects who do become infected and compared with those infected in the absence of vaccination. This would allow one to determine if the vaccine indeed lowers the virus set point.

Trials to prevent EBV-associated malignancies would be difficult but not impossible. An initial trial might try to prevent development of EBV post-transplant lymphoproliferative disorder or to prevent the rapid rise in EBV viral loads that are associated with an increased risk of EBV lymphoproliferative disorder^45, 46^ Most cases of EBV post-transplant lymphoproliferative disorder occur during the first year after hematopoietic stem cell transplantation and during the first 3 years after solid organ transplantation; therefore a lengthy trial might not be needed. A major concern with such a trial is that the transplant recipient would be immunocompromised owing to chronic disease before transplant and would likely be receiving immunosuppressive drugs after transplant. Nonetheless, as the risk of post-transplant lymphoproliferative disorder is much higher in seronegative transplant recipients a vaccine that induced immunity comparable to natural infection might still reduce the rate of disease.

A trial to prevent Burkitt lymphoma in sub-Sahara Africa would be feasible, although it would require a large number of children with extensive coordination. In endemic areas, the median age of patients with Burkitt lymphoma is 8 years, so the time between vaccination and follow-up for development of disease is not prohibitive. In 1978, Guy de-Thé *et al.*^47^ performed a prospective serologic study of 42 000 African children and found that the level of EBV VCA (viral capsid antigen) antibody was significantly higher in the 14 children who subsequently developed Burkitt lymphoma compared with the controls. One difficulty with a vaccine study is that ~50% of African children are infected with EBV by the age of 1 year, and it might be difficult to give two or three doses of vaccine at an early age. Nonetheless, hepatitis B vaccine is given at birth, 1–2 months and 6 months of age, and rotavirus, inactivated poliovirus, Haemophilus influenza type B, diphtheria–tetanus–acellular pertussis and pneumococcal conjugate vaccines are given routinely at 2, 4 and 6 months of age in the United States.

Approximately 1 in 800 persons in Denmark and Sweden with IM developed Hodgkin lymphoma at a median of 4 years after the onset of IM.^5^ Thus, an effective EBV vaccine that prevents IM might reduce the rate of Hodgkin lymphoma. In a study of college students at the University of Minnesota, 37% of freshmen were EBV seronegative.^2^ During a median 3-year follow-up, 36% of the EBV-seronegative students developed IM. Therefore, it might be possible to vaccinate a large group of EBV-seronegative college freshmen and evaluate the rates of IM and Hodgkin lymphoma.

It would be very difficult to show the direct efficacy for a prophylactic vaccine to prevent gastric or nasopharyngeal carcinoma owing to the very long period of time between infection and development of the disease. More effective biomarkers that could predict the development of these diseases at a much younger age would be needed to make vaccine trials for these carcinomas feasible. Boys with X-linked lymphoproliferative disease type 1 are at very high risk of fatal IM and might be candidates for an EBV vaccine. As IM has been associated with a twofold increased risk of multiple sclerosis in several studies,^11^ an EBV vaccine that reduces the risk of IM might also reduce the incidence of multiple sclerosis. The rate of multiple sclerosis varies by geographic area; in the United States about 1:1000 persons develop the disease, so a large number of EBV-seronegative persons would need to be vaccinated to show the effect of the vaccine.

## Therapeutic vaccines

Induction of cellular immunity is the primary goal of therapeutic vaccines for EBV-associated diseases. These diseases include EBV-positive nasopharyngeal carcinoma, Hodgkin lymphoma, non-Hodgkin lymphoma and T-cell lymphoma, which generally have a type 2 EBV latency pattern with expression of EBV EBNA-1, LMP1 and LMP2. EBV-positive gastric carcinoma usually has a type 1 latency pattern with expression of EBNA-1. EBNA-1 is the principle target of CD4 T cells, although LMP1 and LMP2 are also targeted by CD4 cells. In contrast, of the genes expressed in type 2 latency LMP2 is the major target followed by EBNA-1.

A proof of principle for therapeutic vaccines is provided by studies showing that infusions of EBV-specific T cells have activity for Hodgkin lymphoma, non-Hodgkin lymphoma and nasopharyngeal carcinoma. EBV-specific cytotoxic T lymphocytes (CTLs) were generated from 9 of the 13 patients with Hodgkin lymphoma and infusions of cells into three patients resulted in lower EBV DNA loads in the blood of two patients and clinical responses in two patients.^48^ More recently, autologous CTLs specific for LMP2 or for LMP1 and LMP2 were given as adjuvant therapy to 29 high-risk patients or those with multiple relapses with Hodgkin or non-Hodgkin lymphoma; 28 remained in remission at a median of 3 years after receiving the cells.^49^ Similarly, a study of 10 patients with advanced nasopharyngeal carcinoma treated with autologous EBV-specific CTLs resulted in four remissions, including complete responses in two patients who had had refractory disease.^50^ Smith *et al.*^51^ treated patients with metastatic nasopharyngeal carcinoma with EBV-specific autologous T cells; the median overall survival was 523 days in patients who received the cells compared with 220 days in those who did not receive T cells. A subsequent study in which 35 patients with nasopharyngeal carcinoma were treated with chemotherapy followed by EBV-specific CTLs resulted in a response rate of 71%, with two- and three-year survival rates of 63% and 37%, respectively, which were better than a former chemotherapy trial.^52^ Thus, a vaccine that induces T-cell responses to EBV latency proteins expressed in the tumor might enhance survival.

As noted above, many studies suggest that EBV may have an important role in multiple sclerosis. A recent case report described a patient with progressive multiple sclerosis who received autologous virus-specific T cells and had an improvement in clinical, radiologic and immunologic parameters.^53^ If these findings can be duplicated in controlled clinical trials, a vaccine that induces EBV-specific T-cell responses might be used for the treatment of multiple sclerosis provided it could be shown to be safe.

Two types of therapeutic vaccines have been tried for patients with nasopharyngeal carcinoma. Taylor *et al.*^54^ expressed a fusion protein containing the carboxyl terminus of EBNA-1 (important for virus-specific CD4 T cells) fused to LMP2 (important for EBV-specific CD8 T cells) in a poxvirus vector (modified vaccinia Anakara). They showed that dendritic cells infected with the recombinant poxvirus reactivated LMP2-specific CD8 T cells and EBNA-1 specific memory T cells *in vitro* using peripheral blood mononuclear cells from healthy seropositive persons. Hui *et al.*^55^ vaccinated patients in Hong Kong with nasopharyngeal carcinoma in remission with the same vaccine and detected increased T-cell responses to at least one viral protein in 15 of 18 patients and a three to fourfold increase in the magnitude of T-cell responses to the proteins. A follow-up study in the United Kingdom with the same vaccine showed that 8 of the 14 patients tested had increased CD4 and CD8 T-cell responses after vaccination to one or both EBV proteins.^56^

In a second approach, autologous dendritic cells were incubated with EBV peptides or infected with viral vectors expressing EBV proteins and then injected into patients. Lin *et al.*^57^ used autologous dendritic cells incubated with LMP2 peptides to vaccinate patients with nasopharyngeal carcinoma; after four injections CD8 T-cell responses to LMP2 were induced by the peptides, which correlated with tumor regression in two of nine patients. Chia *et al.*^58^ transduced autologous dendritic cells with an adenovirus vector expressing a truncated form of LMP1 and full length LMP2; injection of 16 patients with metastatic nasopharyngeal carcinoma with the dendritic cells resulted in a partial response in one patient and stable disease in two patients, although no expansion of LMP1 and LMP2-specific T cells was detected *in vivo*.

## Conclusions

At a meeting convened at the National Institutes of Health in Bethesda, Maryland in February 2011, the participants agreed for the need of EBV vaccines to prevent IM and the nearly 200 000 cases of EBV-associated malignancies that occur each year in the world.^59^ A number of additional studies were recommended that could facilitate the development and use of EBV vaccines including (a) determining correlates of protection against EBV infection and disease, (b) discovery of surrogate markers to identify persons at a high risk for EBV-associated cancers and (c) epidemiologic studies to better quantify the economic and societal benefit of a vaccine that reduces IM and EBV-associated cancers. Although vaccines are licensed to prevent hepatitis B virus and human papilloma virus-associated malignancies, it is time for the development and approval of a vaccine to prevent EBV-associated diseases.

## Figures and Tables

**Table 1:** Non-human primate studies with EBV gp350

*Vaccine*	*Adjuvant*	*Study and results*
Purified native gp350	Lipsomes	Cotton top tamarins protected from EBV- induced lymphoma^29^
Purified native gp350	ISCOMs	Cotton top tamarins protected from EBV- induced lymphoma^60^
Purified native gp350	Muramyl dipeptide in squalene	Cotton top tarmarins protected from EBV- induced lymphoma^61^
Adenovirus-gp350	None	Cotton top tamarins protected from EBV- induced lymphoma^30^
Vaccinia-gp350 WR strain	None	Cotton top tamarins protected from EBV- induced lymphoma^31^
Vaccinia-gp350 Wyeth strain	None	Cotton top tamarins not protected from EBV- induced lymphoma^31^
Vaccinia-gp350	None	Common marmosets had decreased virus replication of EBV after challenge^62^
Recombinant gp350	Muramyl dipepide in squalene	Cotton top tamarins protected from EBV- induced lymphoma^63^
Recombinant gp350	Alum	Cotton top tamarins protected from EBV- induced lymphoma^64^
Recombinant gp350	Alum	Common marmosets had decreased virus replication of EBV after challenge^65^

Abbreviations: EBV, Epstein–Barr virus; ISCOMs, immunostimulating complexes WR strain, Western reserve strain.

**Table 2:** Human trials of EBV vaccines

*Vaccine*	*Adjuvant*	*Results*
*Prophylactic*		
Vaccinia-gp350	None	Induced neutralizing antibody and may have reduced infection^35^
Recombinant gp350	None, alum, or alum/MPL	Induced neutralizing antibody^36^
Recombinant gp350	Alum/MPL	Induced neutralizing antibody; reduced rate of infectious mononucleosis, but not infection^37^
Recombinant gp350	Alum	Induced transient neutralizing antibody in a minority of patients with chronic kidney disease; did not prevent EBV post-transplant lymphoproliferative disorder^38^
EBNA-3A peptide	Tetanus toxoid in oil and water emulsion	Trend toward reduction of infectious mononucleosis but not infection^39^

*Therapeutic*		
Modified vaccinia Ankira expressing LMP2 and a portion of EBNA-1	None	T-cell responses to LMP2 or EBNA-1 detected in 15 of 18 NPC patients with a three to fourfold increase in T-cell responses^55^
Modified vaccinia Ankira expressing LMP2 and a portion of EBNA-1	None	T-cell responses to LMP2 or EBNA-1 detected in 8 of 14 NPC patients^56^
Autologous dendritic cells pulsed with LMP2 peptides	None	Boosted CD8 T-cell responses to LMP2 in NPC patients; tumor regression observed in two of nine patients^57^
Autologous dendritic cells transduced with adenovirus expressing LMP2 and a portion of LMP1	None	Induced LMP-specific delayed type hypersensitivity responses in 75% of NPC patients, but no increase in LMP1 or LMP2-specific T cells; transient partial response in 1 of 16 patients, and stable disease in 2^58^

Abbreviations: EBNA, Epstein–Barr virus nuclear antigen; EBV, Epstein–Barr virus; LMP1, latent membrane protein 1; MPL, monophosphoryl lipid A; NCP, nasopharyngeal carcinoma patients.
